# Emerging Mechanisms of Insulin-Mediated Antiviral Immunity in *Drosophila melanogaster*

**DOI:** 10.3389/fimmu.2019.02973

**Published:** 2019-12-20

**Authors:** Chasity E. Trammell, Alan G. Goodman

**Affiliations:** ^1^School of Molecular Biosciences, College of Veterinary Medicine, Washington State University, Pullman, WA, United States; ^2^NIH Biotechnology Graduate Training Program, Washington State University, Pullman, WA, United States; ^3^Paul G. Allen School for Global Animal Health, College of Veterinary Medicine, Washington State University, Pullman, WA, United States

**Keywords:** innate immunity, RNA interference, JAK/STAT, insulin, STING, West Nile virus, Zika virus, dengue virus

## Abstract

Arboviruses (arthropod-borne viruses), such as Zika (ZIKV), West Nile (WNV), and dengue (DENV) virus, include some of the most significant global health risks to human populations. The steady increase in the number of cases is of great concern due to the debilitating diseases associated with each viral infection. Because these viruses all depend on the mosquito as a vector for disease transmission, current research has focused on identifying immune mechanisms used by insects to effectively harbor these viruses and cause disease in humans and other animals. *Drosophila melanogaster* are a vital model to study arboviral infections and host responses as they are a genetically malleable model organism for experimentation that can complement analysis in the virus' natural vectors. *D. melanogaster* encode a number of distinct mechanisms of antiviral defense that are found in both mosquito and vertebrate animal systems, providing a viable model for study. These pathways include canonical antiviral modules such as RNA interference (RNAi), JAK/STAT signaling, and the induction of STING-mediated immune responses like autophagy. Insulin signaling plays a significant role in host-pathogen interactions. The exact mechanisms of insulin-mediated immune responses vary with each virus type, but nevertheless ultimately demonstrates that metabolic and immune signaling are coupled for antiviral immunity in an arthropod model. This mini review provides our current understanding of antiviral mechanisms in *D. melanogaster*, with a focus on insulin-mediated antiviral signaling, and how such immune responses pertain to disease models in vertebrate and mosquito species.

## Introduction

Mosquitoes are a prominent vector for various arboviruses including West Nile virus (WNV), Zika virus (ZIKV), and dengue virus (DENV). These viruses pose a significant concern to human populations as the mosquitoes' continual encroachment into previously unexposed regions expands (1, 2). This habitat expansion renders more individuals at risk of exposure with limited, if any, treatments available. Climate change has also resulted in alterations in mosquito seasonal activity (3) and feeding behavior (4) resulting in increasing frequency and severity of arboviral cases. There is a direct correlation between the expansion of vector-competent mosquitoes and disease incidence within afflicted regions [reviewed in (5, 6)] indicating that vector activity is a significant risk factor for arboviral disease.

**Figure 1:**
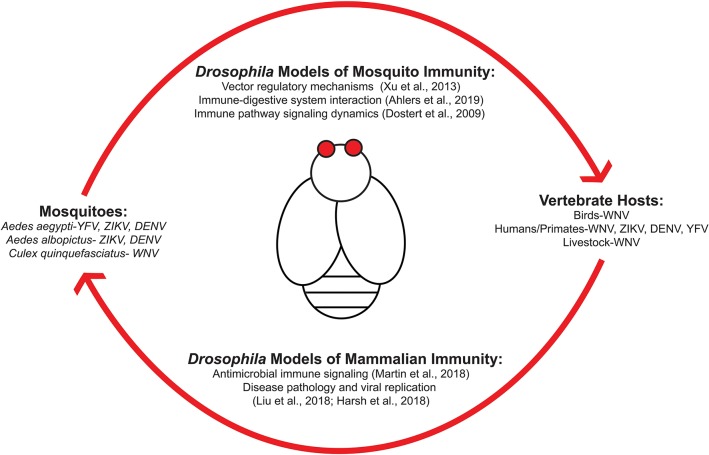
*Drosophila melanogaster* are an ideal model organism for studying host-arboviral interactions. Various arboviruses utilize mosquitoes as reservoirs and vectors for transmission into vertebrate hosts. This can include species that are either involved in viral replication and spread (such as bird populations for West Nile virus) or dead-end host that become infected without being able to properly propagate viral replication for further spread (i.e., humans). Transmission is accomplished via a bloodmeal exchange. *Drosophila* possess orthologous host response pathways found in mosquitoes and humans, making it an ideal model organism for studying transmission dynamics and host-pathogen interactions at both vector and human level.

Figure 1 outlines the transmission cycle of various arboviruses as they move within host populations and how *Drosophila melanogaster* can be used to study arboviral immunity for each system. Transmission from mosquito to vertebrates requires a bloodmeal exchange where infected saliva is ejected into the new host. Viral replication then permits the spread of virus from infected host to mosquito to continue the transmission cycle (Figure 1). Research is required to identify the signaling responses used in regulating these viruses at the vector and human level. Studies regarding immune responses initiated during the initial bloodmeal exchange (7) are important as this event is a key determinant whether transmission occurs (8). An emphasis as to how immune and nutritional signaling interact with one another is of particular interest as both would be active during ingestion of an infected bloodmeal. Insulin-mediated signaling regulates numerous viruses by inducing activation of canonical immune pathways (9). Because insulin is ingested during the bloodmeal, a recent study has shown that vertebrate insulin is able to regulate the type of innate immune response that occurs during viral infection in insect vector hosts (10).

Previous work has identified the signaling pathways that respond to arboviral infection and their significance with respect to disease outcome and severity (11–13). *Drosophila* have proven to be a significant model organism for studying arboviruses as many of the signaling pathways identified are conserved amongst insect species [reviewed in (14)]. These studies have utilized the genetic power provided by the *Drosophila* system to demonstrate the effect that nutritional status poses on host immunity.

Immune responses during various arboviral infections are evolutionarily conserved among insects and include the canonical RNA interference (RNAi) (13, 15, 16), JAK/STAT (10, 12), and STING-mediated signaling (17). These pathways are associated with the insulin/insulin-like growth factor signaling (IIS) pathway and have been established as key determinants in vector competency and disease outcome (9, 18). It has been demonstrated that ingestion of vertebrate insulin regulates whether an RNAi- or JAK/STAT-mediated response is active during infection against WNV (10). STING-mediated immunity has been previously linked to induce JAK/STAT signaling (19) and affects nutritional homeostasis during infection (20, 21) implying that it may be regulated by insulin as well. Since insulin-mediated signaling appears to have a broad impact on insect immunity, recent studies have sought to establish how insulin connects each antiviral pathway to respond to different arboviruses. Because vector competency and transmission is so closely dependent on gut-associated immune signaling, the connection that nutrition and immunity has is indicative of their importance in regulating infection (9, 22). This mini review presents a condensed understanding regarding the major responses that occur during arboviral infection using *Drosophila*, the role that insulin signaling plays, and a summation of current and future efforts taken within this field.

## RNA Interference Pathway

**Figure 2:**
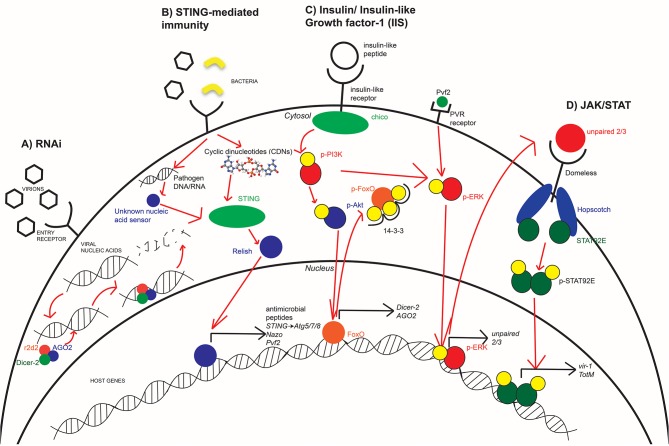
Innate immune antimicrobial pathways are conserved in arthropods. Insects utilize RNAi **(A)**, STING-mediated immunity **(B)**, and JAK/STAT signaling **(D)** in order to effectively respond to various arboviruses at different stages of infection. The IIS pathway **(C)** is an important mediator in host immunity as it regulates which immune responses are active or suppressed. During times of starvation, RNAi is more active while the bloodmeal provides the needed insulin to suppress RNAi and activate JAK/STAT. STING-mediated immunity has not yet been directly linked to IIS but may be affected by broader nutritional signaling. Each of these pathways, to varying degrees, are conserved in fly, mosquito, and human systems with different efficiencies in responding to viral infection.

One of the most broadly restricting antiviral responses used by insects is the RNAi pathway (13, 16). RNAi signaling occurs in response to detection of viral nucleic acids within the cytosol of infected cells. In *Drosophila*, recognition of viral nucleic acids by the endonuclease Dicer-2 results in the recruitment of proteins Argonaute-2 (AGO2) and r2d2 to form an RNA-induced silencing complex (RISC) (13, 23). This results in the cleavage and degradation of bound viral nucleic acids (13, 23, 24). While this antiviral response is a significant component of insect immunity against RNA viruses like Sindbis virus (SINV) (24) and ZIKV (25), an arthropod-borne alphavirus and flavivirus, respectively, RNAi has also been shown to respond to DNA viruses like Invertebrate iridescent virus 6 (IIV-6) (26) (Figure 2A). While Harsh et al. showed that the loss of the RNAi component, Dicer-2, resulted in increased ZIKV replication and mortality, they linked the increased susceptibility of these flies to dysregulated homeostasis of the gut and fat body. Moreover, studies have shown that another RNAi component, namely AGO2, is dispensable for an antiviral response against ZIKV (17, 25). Additionally, there are flaviviruses that encode viral suppressors of RNAi (VSR) (27). Not only do VSRs function in *Drosophila* model systems (28), but they also function during WNV, DENV, and Yellow fever virus infections in *Culex* mosquitoes (29, 30). Thus, the antiviral role of RNAi depends not only on the host but also virus type. Further studies are needed to clarify the antiviral role of RNAi in model and vector organisms, especially with respect to flaviviruses and the VSRs they may encode. Mosquitoes become infected and spread disease via bloodmeal exchanges. Because of the direct role that nutritional acquisition has during infection, its role in antiviral immunity is mediated in part by regulating RNAi. In both insect and mammalian systems, IIS regulates the transcription factor forkhead box O (FOXO), and FOXO is predominately associated with longevity and nutritional signaling as it induces *dInR* (*insulin-like receptor*) in *Drosophila* (31, 32). FOXO possesses a secondary role in host immunity through its induction of RNAi-specific genes. Specifically, FOXO regulates the transcription of *Dicer-2* and *AGO2* in *Drosophila* and is demonstrated to enhance RNAi signaling during Cricket paralysis viral infection (18). Since IIS regulates FOXO transcriptional activity, there is a direct connection between RNAi immunity and insulin signaling. In *Drosophila*, the IIS pathway is induced by insulin-like peptides (ILPs) binding to dInR (33). Upon binding, a phosphorylation cascade commences that includes phosphorylation of PI3K and Akt. This results in the phosphorylation of nuclear FoxO at three residues, its association with the 14-3-3 chaperone protein, and export into the cytosol (32, 34) (Figure 2C). Insulin treatment is demonstrated to result in transcriptional suppression of *Dicer-2* and *AGO2* to reduce RNAi signaling (10). This insulin-mediated suppression of RNAi proposes that mosquitoes have evolved multiple immune responses to pathogens that is dependent on its nutritional status.

RNAi is evolutionarily conserved across organisms; however, its role in antiviral immunity varies between insects and mammals. While the signaling cascade and proteins involved are conserved, mammals have evolved other sensing mechanisms to detect and respond to viruses. RIG-I and MDA5 RNA sensing are canonical pathways for vertebrate innate immunity and have developed from RNA sensors like Dicer (35). In particular, RIG-I and MDA5 are shown to be critical immune regulators in response to flaviviruses like ZIKV (36) and WNV (37) and alphaviruses like SINV (38). This would imply that while RNAi is still fully functional in mammals, its role in responding to viruses is less stringent than in insect systems [reviewed in (39, 40)]. Other invertebrate and plant species utilize RNAi signaling as a means of antiviral immunity [reviewed in (41, 42)]. Researchers interested in studying RNAi immunity against arboviruses utilize *Drosophila* as the signaling cascades described are well-conserved in the more relevant mosquito vector. While the functional antiviral role of RNAi varies in mammalian systems, the proteins and signaling events involved are conserved. Because of this, studies utilizing *Drosophila* have proven it to be a viable model for study in host immunity and other regulatory functions.

## JAK/STAT Pathway

Innate immunity uses various responses to different viruses. These responses include phagocytosis of viral particles or by inducing production of downstream cytokines and antiviral effectors (43). Whereas, the RNAi pathway provides a broad means of protection through the degradation of viral nucleic acids (16), the JAK/STAT pathway is a signaling cascade where detection of infection or other stimuli results in the induction of antiviral effectors like *vir-1, Vago*, and *TotM* (12, 24, 44). In *Drosophila* immunity, this pathway is activated upon detection of infection (45), resulting in induction of the *unpaired* ligands (46, 47). Unpaired ligands bind to the receptor domeless (48), resulting in the Janus kinase (JAK) ortholog hopscotch to be phosphorylated and form docking sites for phosphorylation and dimerization of the STAT92E transcription factor (12, 49). The activated STAT92E protein complex is imported into the nucleus to induce transcription of downstream antiviral effectors. This includes *TotM* during early stages of infection (24) and *vir-1* during later stages (12) (Figure 2D). JAK/STAT has been shown to be involved in the immune response against the insect virus *Drosophila* C virus but not SINV (24) in *Drosophila*, and JAK/STAT is antiviral against WNV in both *Drosophila* and *Culex* mosquitoes (10, 50).

The connection between JAK/STAT and IIS is not as direct as insulin's effect on the RNAi pathway, but recent research has demonstrated that insulin signaling in insects controls a switch between RNAi- and JAK/STAT-dependent responses. Specifically, when insulin treatment causes transcriptional suppression of RNAi, insects induce enrichment of JAK/STAT (10). This is mediated by insulin's phosphorylation and activation of downstream Akt and ERK proteins during SINV and DENV infection (9, 22) that was further evaluated to induce immunity through JAK/STAT against WNV (10). Insulin-mediated induction of JAK/STAT then induces the transcription of downstream antiviral effector proteins *vir-1* and *TotM* (10, 12, 24). JAK/STAT immunity responds to pathogens during early stages of infection in the mosquito which corresponds to ingestion of an infected bloodmeal and escape from the midgut to distal tissues (51). This association between IIS and JAK/STAT signaling is indicative that vector-competent insects have evolved immune mechanisms that are responsive to nutritional acquisition. While mosquitoes use an RNAi-dependent response during times of starvation, the bloodmeal provides the insulin needed to activate a JAK/STAT-dependent response during infection. While the ability to regulate immune responses based on nutritional status and how it impacts viral efficacy and transmission has yet to be established, it is plausible that insulin-mediated signaling could be targeted in future vector-control protocols. Its potential as a target depends on whether insulin's antiviral activity in the salivary glands is just as important as it is in midgut or fat body, critical digestive and immune organs (8, 9).

JAK/STAT signaling is an evolutionarily conserved immune response utilized in both insect and mammalian systems. Induction of JAK/STAT in mammals results in a Type I interferon (IFN) response against viral infection (52). The JAK/STAT pathway is similarly regulated by RIG-I-like receptor (RLR) signaling in mammalian systems (35, 53). This pathway is an important means of responding to WNV in both insects (10, 44) and mammals (52, 53). Because JAK/STAT is related to other regulatory processes like cell proliferation and differentiation [reviewed in (54)], researchers have also used the *Drosophila* model to study the pathway's role in a non-immunological context like cellular growth (47), differentiation (48), polarization (49), and oogenesis (55). *Drosophila* provide a unique system for studying JAK/STAT signaling at various levels of complexity in both an immune and regulatory context to provide clarity regarding the pathway's significance in the organism.

## Sting-Mediated Immunity

Mediators of mammalian immunity have evolved from established signaling pathways that are present in invertebrate systems (35, 56). One such set of responses is stimulator of IFN genes (STING)-mediated immunity. Upon detection of viral nucleic acids in the cytosol, the DNA sensor cGAS metabolizes cyclic dinucleotides that bind to and activate STING (11), which induces phosphorylation and activation of various transcription factors like TBK1, IRF3, and STAT6 (19, 57). These transcription factors regulate the induction of Type I IFN responses through the secretion of IFN-α and β (11, 19, 57). STING-mediated immunity has been heavily studied within mammalian systems; however, it is only recently that STING and its role in innate immunity have been identified in insects and other invertebrates (17, 56, 58–60) (Figure 2B).

In *Drosophila*, STING signaling provides immunity against both bacterial and viral infections. During infection with *Listeria monocytogenes*, cyclic dinucleotides are produced which results in STING-mediated signaling and nuclear import of Relish, the fly ortholog of mammalian NF-κB (56). This immune response induces transcription and secretion of IMD-characteristic antimicrobial peptides to reduce bacterial burden (56). STING-mediated antiviral immunity also occurs through Relish and IKKβ, which regulate expression of the antiviral factor *Nazo* (58). In the silkmoth, *Bombyx mori*, STING signaling activates antiviral activity through Dredd and IMD, leading to Relish signaling and induction of antimicrobial peptides against nucleopolyhedrovirus (NPV) (59). Other studies using the *Drosophila* system have further evaluated STING-mediated immunity by autophagy (17).

Autophagy is a cellular process in which intracellular structures and proteins are degraded in a lysosomal-dependent manner [reviewed in (61)]. Because viruses are obligate intracellular pathogens, autophagy is an established antiviral response that is partially regulated by nutritional and STING-mediated signaling (62). While STING-mediated autophagy has been established in responding to numerous viruses in mammals, recent studies using *Drosophila* have demonstrated that insects can utilize autophagy to respond to ZIKV in neuronal tissues (17). Specifically, ZIKV infection results in a pro-inflammatory response in *Drosophila* brains which induces STING-mediated activation of autophagy and immunity (17). This form of antiviral immunity is indicative that neuronal protection against arboviruses is mediated through STING-mediated signaling. This study provides another example of the versatility provided in using the *Drosophila* system as the fly model is a viable means for studying antiviral immunity conserved between invertebrates and vertebrates.

Unlike the RNAi and JAK/STAT pathways, which are both regulated in part by insulin signaling, a direct link between IIS and STING has yet to be shown. However, since autophagy is partially regulated by nutritional status [reviewed in (63)] and STING has been previously shown to induce *STAT6* in the mammalian system (19), it is plausible that STING-mediated immunity may also be partially regulated by insulin signaling. Since STING has only recently been discovered in the insect model, future research is needed to further evaluate how STING connects to other canonical immune and nutritional pathways and its involvement in vector competency.

## Prospective

Both the Centers for Disease Control and Prevention (CDC) and World Health Organization (WHO) agree that mosquito-borne arboviruses will be of great concern in the following years due to the expansion of mosquitoes' habitation range and activity into previously unexposed regions (64, 65). Research using *Drosophila* have permitted investigators to identify the key signaling events that occur during infection and develop more effective vector control protocols that target viral replication and likelihood of transmission. Recently, *Drosophila* have been used to identify ingestion of mammalian insulin as a key regulator in controlling WNV replication in the insect model. The genetic screen to identify insulin receptor was performed using *Drosophila*, and the role of insulin signaling was then validated in the mosquito model (10).

While *Drosophila* have been an invaluable tool in the study of arboviruses in place of the more relevant arthropod vector, there are limitations. For example, JAK/STAT signaling, while protective in the mosquito model against arboviruses such as WNV and DENV (10, 44, 66), may not be protective in *Drosophila* during SINV or vesicular stomatitis virus infections (12, 24). Because *Drosophila* are not the natural host for these arboviruses, there are limitations surrounding whether the responses observed are indeed what occurs in natural hosts. As such, work involving *Drosophila* must ensure that their findings are further evaluated in the more relevant model, whether that be the insect vector or human host.

Developing disease response protocols that aim at preventing transmission from arthropods to humans would be the most beneficial in terms of cost-efficiency and alleviating disease burden at a global scale. Research efforts also aim to identify novel therapeutics that are effective at treating humans post-exposure. Because many of the immune pathways discussed here are also present in humans (Figure 2), future research aimed at identifying novel human-specific antiviral therapeutics could benefit from the use of *Drosophila*. The use of *Drosophila* to model host immunity at both the mammalian and vector level during arboviral infection has provided a greater depth of knowledge regarding which signaling pathways are involved during infection and how they can be targeted in the arthropods that transmit disease.

## Author Contributions

CT wrote the first draft of the manuscript. AG revised the manuscript.

### Conflict of Interest

The authors declare that the research was conducted in the absence of any commercial or financial relationships that could be construed as a potential conflict of interest.
